# Seeing Anxiety: Ecological Video Ethnography and Simulation to Understand Anxiety and Decision‐Making in Dental Care

**DOI:** 10.1111/hex.70687

**Published:** 2026-05-14

**Authors:** Mona Nasser, Hannah Drayson, Sally Hanks, Nicholas Peres, Michael Punt

**Affiliations:** ^1^ Peninsula Dental School, Faculty of Health University of Plymouth Plymouth Devon United Kingdom; ^2^ School of Art, Design and Architecture, Faculty of Art, Humanities and Business University of Plymouth Plymouth Devon United Kingdom; ^3^ Torbay and South Devon NHS Foundation Trust Torquay Devon; ^4^ Centre for Arts in Health Falmouth University Falmouth Cornwall United Kingdom

**Keywords:** affect theory, clinical training, dental anxiety, ecological analysis, non‐verbal communication, patient experience, simulation, video ethnography, video reflexive ethnography

## Abstract

**Background:**

Dental anxiety is a widespread barrier to care, often contributing to appointment avoidance, treatment disruption, and emotional strain for both patients and clinicians. While extensively studied as a psychological trait, less attention has been paid to how anxiety manifests situationally and relationally, through non‐verbal, affective, and spatial cues within clinical encounters. Moreover, in certain contexts, such affective signals can intersect with decision‐making processes, shaping how patients interpret, engage with, or withdraw from clinical choices.

**Objective:**

To explore how anxiety manifests within the micro‐choreography of dental encounters, develop a taxonomy of ‘anxious tells’, and assess the utility of ecological video ethnography and simulation‐based camera testing in supporting affect‐aware training and potential AI integration.

**Methods:**

We conducted a two‐phase qualitative study. Phase one involved re‐analysis of over 70 h of ethnographic video from dental clinics, guided by threshold theory, affect theory and ecological‐relational analysis. Phase two consisted of simulation‐based testing of multi‐angle camera setups in a custom‐built dental lab designed to optimise audiovisual capture of non‐verbal cues.

**Results:**

Anxiety was expressed through posture shifts, facial micro‐expressions, breath modulation, eye movement, and interactional hesitancy. These behaviours were often relational and environmentally cued, and in some cases intersected with moments of decision‐making such as consent, treatment planning, or pain management. We developed a context‐sensitive taxonomy of ‘anxious tells’, identified optimal camera protocols, and revealed interpretive blind spots through interdisciplinary co‐viewing. Key challenges included managing large AV data, synchronisation, and ethical clarity in consent.

**Conclusion:**

This study demonstrates how video can function not only as an observational device but as a collaborative instrument for clinical reflection, training, and research co‐production. It is useful to conceptualise anxiety in dentistry not as a fixed trait, but as a relational and affective phenomenon. The newly developed taxonomy of ‘anxious tells’ in dental practice supports the identification and effective clinical care of patients suffering from such anxiety. Ecological video ethnography offers a powerful lens for revealing these dynamics and supporting situated, affect‐based training. Our framework and toolkit lay the groundwork for future human‐in‐the‐loop AI systems that recognise, contextualise, and respond to affective cues supporting safer, more empathetic care.

**Patient or Public Contribution:**

As part of an EPSRC IAA grant, internal strategic investments, and an MPS grant, we conducted a series of patient and public engagement activities that shaped both the research question and the study design. We also ran sessions with dentists. The PPIE process directly informed the consent form, as participants highlighted that some individuals might want their data to be made available while others may not. A key divide emerged: while some were comfortable with filming, others were strongly opposed. Contrary to our expectations, most dentists were supportive of the idea. During the sessions, we also learned that some dentists routinely record patient interactions for potential legal purposes, which highlighted an additional potential use case for our toolkit.

## 1 Introduction

Dental anxiety in patients remains a significant barrier to accessing and delivering quality oral healthcare. It contributes not only to their avoidance of routine treatment and late presentations but also to heightened stress for both patients and clinicians during procedures, with implications for clinical outcomes and professional burnout [1, 2, 3]. While numerous studies have investigated dental anxiety from psychological and behavioural perspectives, the subtle, dynamic, and relational nature of anxiety in the dental clinic remains underexplored, particularly through a sensory, ecological lens [1, 2, 3].

Traditional research on dental communication has focused primarily on verbal exchanges. Yet, clinical encounters are saturated with non‐verbal signals—gestures, postures, micro‐movements, changes in breathing, tonal shifts, and even silence that reveal the affective state of both patient and dentist [4, 5, 6]. The term *affective dynamics* describes these embodied, often pre‐conscious responses that can shape the clinical interaction; and *anxious tells* refers to the noticeable subtle, non‐verbal, context‐dependent expressions of these responses that fluctuate within encounters.

In recent years, video ethnography has gained traction as a valuable method for examining the nuanced dynamics of clinical interactions. Yet, its use in dentistry remains limited, often constrained by rigid observational protocols or suboptimal recording and playback setups [7, 8]. Existing studies tend to fragment affective communication into discrete, quantifiable units, overlooking its emergent, non‐linear nature. A recent pilot study applied a multisensory analytical framework to recordings of child–parent–dentist encounters, revealing that tailored environmental modifications—spanning visual, auditory, and somatosensory dimensions—can effectively alleviate child anxiety [9]. Building on these insights, we piloted an ecological‐relational approach to video ethnography [10], one that foregrounds the entangled relationships between people, spaces, sounds, tools, and atmospheres in the co‐creation of anxiety (patients and dental staff) in the dental environment.

Patient and Public Involvement and Engagement (PPIE) was embedded as a constitutive element of this study's design rather than a later downstream consultation activity. Early PPIE engagement shaped not only ethical thresholds around filming but also core methodological decisions, including consent models, acceptable uses of video data, and the boundaries of what participants considered legitimate observation.

This study supports a richer understanding of patient anxiety and contributes practical innovations to support future research, education, and clinical reflection. Such innovations include a taxonomy of non‐verbal cues of patient anxiety; a framework for anxiety‐responsive care education; and guidance for successful camera placement for recording in dental practice.

## 2 Research Objective

Through exploring the dynamic, contextual, and relational dimensions of dental anxiety using a novel ecological‐relational approach to video ethnography, and drawing on 79 h of dental procedure footage plus data from a simulation lab, the study aims to answer the research question:

*How does dental anxiety manifest through embodied, relational, and environmental cues within clinical encounters, and how can ecological video ethnography make these dynamics visible for research and training?*

Our primary objective was:

1. **Identify and classify non‐verbal ‘tells’ of anxiety** in patients, including body movements, facial expressions, breathing patterns, and micro‐interactions with their surroundings.
  Our secondary objectives were:
2. **Assess the strengths and limitations of video ethnography** in capturing affective dynamics in clinical care, and its potential application in training and reflective practice.
3. **Develop a practical guide for camera placement and use** in both clinical and simulated dental settings, supporting high‐quality, ethically sound video data collection for anxiety‐related research and training.

## 3 Methods

### 3.1 Study Design Overview

A two‐phase, multi‐method qualitative design approach explored how dental anxiety manifests and evolves within clinical encounters. Our approach integrates video ethnography with simulation‐based research to develop both theoretical insight and practical tools for training and practice.

The first phase involved the secondary analysis of over 70 h of video recordings from routine dental procedures, originally collected under a prior ethnographic project examining leadership in dental practice. These recordings offered a unique opportunity to investigate humanistic interactions between dentists, patients, and support staff in real‐world conditions. Drawing on an ecological‐relational perspective, the material was reanalysed specifically for overt behavioural cues, affective atmospheres, spatial configurations, embodied gestures, and relational dynamics that contribute to the experience of anxiety in the dental setting. The data helped shape the taxonomy of ‘anxious tells’ (Objective 1) and assess the applicability of video ethnography for studying the ecology of dental anxiety (Objective 2). It also provided initial insights into the need for guidance on camera placement, which informed the development of the third objective.

The second phase complemented the observational material with a simulation‐based study conducted in a purpose‐built dental simulation lab that functioned as a liminal learning environment. This setting allowed us to systematically test different video capture setups, refine observational protocols, and develop a practical guide for recording dental encounters in a way that sensitively captures affective, non‐verbal communication. We conceptualised the simulation lab as a boundary space where practitioners could safely encounter ‘troublesome knowledge’ Meyer and Land [11], in this case, the realisation that anxiety is not ‘in’ the patient but emerges relationally through clinical encounters. This step was central to addressing Objective 3 and additionally provided further insights and a validity check for Objectives 1 and 2.

Taken together, these two phases formed an iterative loop: ethnographic insights from real‐world practice informed the design and priorities of the simulation phase, while methodological innovations from the simulation lab fed back into how we interpreted and visualised the complexities of anxiety in the clinical footage. This design also allowed us to attend to both theoretical depth and practical application, ensuring the relevance of our findings for dental education, policy, and practice.

### 3.2 Patient and Public Involvement and Engagement (PPIE)

PPIE was embedded throughout the study to inform the design, ethical framing, and acceptable use of video‐based methods in dental settings. PPIE activities included stakeholder discussions with patients, carers, clinicians, educators, and researchers, convened to explore attitudes toward recording clinical interactions, the use of emerging technologies, and experiences of anxiety in dental care.

PPIE contributors expressed diverse and sometimes conflicting views on video recording. While some participants viewed recording as reassuring and potentially protective for both patients and clinicians, others raised concerns regarding privacy, behavioural modification, dignity, and data security. Contributors emphasised the importance of transparency, choice, and clarity regarding how recordings would be used, stored, accessed, and for how long. These discussions directly informed the development of tiered and revisable consent options, enabling participants to opt into different levels of recording and secondary use.

PPIE input also shaped the methodological approach by foregrounding the contextual nature of anxiety. Contributors highlighted that behaviours interpreted as anxiety during treatment may reflect factors external to dentistry, such as prior stress, family circumstances, appointment logistics, or previous healthcare experiences. This reinforced the need for an ecological and relational interpretation of recorded material and informed both the simulation protocol and analytic framework adopted in the study.

PPIE contributors emphasised the importance of discretion and minimal intrusion when introducing recording technologies into clinical environments, particularly for vulnerable groups. These insights informed decisions regarding camera placement, the scope of recording, and the framing of video data as a reflective and educational resource rather than a surveillance tool.

### 3.3 Real‐World Video Data

The 79 h real‐time video footage of dental practice was originally collected during a prior ethnographic project exploring leadership in dental practices, under ethical approvals from the Health Research Authority (IRAS project ID: 162295) and the University of Plymouth Ethics Committee (Reference: 16/17‐698). They were recorded from one angle based on availability of appropriate surfaces. Dental procedures recorded included a diverse range of interventions—from routine fillings and crown replacements to more complex extractions ‐ providing a wide context in which to observe anxiety—related phenomena.

The footage spans different settings and patient demographics, with a significant proportion of participants being older adults receiving care in mostly private practices. These contexts are particularly relevant as older populations are often underrepresented in video‐based health research yet may exhibit distinct forms of affective communication linked to mobility, cognition, and trauma history.

The original camera setups varied across sites but generally provided wide‐angle views of the dental room, allowing for environmental context such as lighting, movement around the chair, or even background music to be captured. However, limitations in some angles particularly occlusions of facial expressions and restricted visibility of fine motor movements became a key impetus for the simulation lab phase, which sought to develop more intentional camera protocols for future studies.

### 3.4 Video Ethnographic Methodology

Our re‐analysis of video footage followed an ecological‐relational methodology, conceptualising anxiety as an emergent property of interactions among bodies, tools, spaces, routines, and atmospheres rather than an individual psychological trait. Drawing on Raymond Williams' concept of structures of feeling [12, 13], the analysis attended to the pre‐conscious affective atmospheres shaping experience and practice in the dental surgery. The guiding analytic question was how anxiety is co‐produced through the choreography of clinical care.

Analysis was informed by thick description [14] and reflexive ethnography, using micro‐contextual questioning, particularly ‘Why that now?’ [15] to examine sequences of action, gaze, gesture, hesitation, and rhythm. Rather than coding static symptoms, we identified dynamic ‘anxious tells’: situated, relational expressions of discomfort that fluctuate within interaction.

The analytic process was iterative and included frame‐by‐frame and segment‐level analysis of routine procedures. Real‐time, fast‐forward, and slow‐motion viewing surfaced temporal patterns; and multi‐modal attention to sight, sound, space, silence, and proximity. These techniques supported fine‐grained analysis of affective dynamics not accessible through real‐time observation alone.

Interpretation was explicitly reflexive, recognising meanings as co‐constructed and situated. The camera was treated as a methodological actor shaping both visibility and interpretation, rather than a neutral recording device [16]. Each viewing constituted an ethnographic encounter shaped by the positionalities of researchers and clinicians, consistent with dialogic approaches to video analysis in medical simulation research [17]. It was conducted through multidisciplinary co‐viewing sessions involving ethnographers, dental clinicians, simulation specialists, artists, and design researchers. This interpretive plurality surfaced differing professional assumptions and attentional habits, rendering visible how the same gesture could be read differently across disciplines. Interpretation was therefore recognised as dialogical and provisional.

Researchers documented affective responses during viewing as part of reflexive memoing, recognising affective transference as analytically informative rather than bias to be eliminated. Interpretation was guided by three reflexive principles: temporal reflexivity (attending to how meaning emerges across time), relational anchoring (interpreting cues only within interpersonal and spatial context), and ethical attentiveness (foregrounding ambiguity and safeguarding participant dignity).

Subtle non‐verbal anxious tells including fidgeting, rigid posture, gaze aversion, and micro‐pauses, were examined relationally in connection with clinicians' movements, procedural flow, patients' sensory fields, and ambient clinic conditions. Video thus functioned as more than a record, enabling cross‐disciplinary interpretation and longitudinal pattern recognition. This methodological approach informed subsequent taxonomy development and simulation‐based refinements.

### 3.5 Camera Testing and Protocol Development

The re‐analysis of the clinical footage highlighted both the potential and the limitations of existing video capture methods. While fixed wide‐angle cameras were useful in documenting overall room dynamics, elements of human factors‐based considerations and interactions, they often failed to capture subtle non‐verbal tells such as changes in facial expression, micro‐gestures, or shifts in breathing and posture. These limitations, combined with the ethical and PPIE preferred imperative to design minimally intrusive but maximally sensitive recording setups, motivated the second phase of this study: the development and testing of camera protocols in a simulated dental environment.

This phase was conducted in a purpose‐built simulation lab, replicating the spatial configuration of a dental surgery, including patient chair, light fixtures, assistant stations, and ambient soundscape. The simulation setting allowed systematic exploration of various camera placements, types, and combinations, while maintaining control over scene variables such as lighting, sound, and movement. The study involved a series of technical simulation set‐ups in Torbay Hospital and Simulation lab by the research team. As part of this, we received ethics approval for University of Plymouth Ethics Committee (Project ID: 4982 FOAHB‐A4982) and ran two sessions of video interactions with simulated patients.

### 3.6 Camera Types and Placements Tested

We trialled multiple camera configurations, each assessed for its ability to capture non‐verbal and relational cues without disrupting the natural rhythm of interaction:

- **Overhead fixed cameras** (e.g., ceiling‐mounted GoPros): provided stable, wide‐angle views of the entire clinical space. These were ideal for mapping spatial choreography, tool use, and overall atmosphere, but limited in capturing facial detail.
- **Vertical Column of the dental chair:** cameras mounted on the vertical column of the dental chair facing the patient.
- **Chair light‐mounted cameras**: cameras mounted on the dental light arm offered an adjustable top‐down view of the patient's face and body. These angles were particularly effective in capturing **facial expressions, eye movement, and upper‐body tension**, and could be aligned with the dentist's own visual field. However, they had a risk of oversaturation ‘blow out’ from the light.
- **Body‐mounted cameras** (e.g., chest‐mounted GoPros worn by dentists or assistants): useful for capturing **first‐person perspectives**, particularly hand movements and tool‐patient interactions. However, these presented challenges in stabilisation and patient consent due to perceived intimacy.
- **Tripod‐mounted side views**: placed at a 45‐degree angle to the chair, these captured both patient and dentist profiles, supporting **interpretation of interactional synchrony** and body mirroring.

Each setup was evaluated based on five key criteria:

1. **Visibility** of key body areas (face, hands, torso).
2. **Audio clarity** and spatial orientation of sound.
3. **Interference** with clinical workflow or participant comfort.
4. **Capacity for multi‐angle synchronisation** during post‐processing.
5. **Adaptability** to real‐world clinical constraints (e.g., room size, infection control, power access).
6. **Acceptability and relative ‘invisibility’ to the patient.**

### 3.7 Consent Procedure

We provided the participants with three options as part of the consent form to provide them with more choice and flexibility around how their data is used.

Option 1: Project outputs. I give permission for the footage of the session and interview to be used by the project team in outputs (academic papers and presentations) and public dissemination of the project.

Option 2: Anonymous analysis. I would prefer for the video footage to be analysed and then deleted. I would not like my audio nor video recordings to be made public in this project.

Option 3: Public research data sharing. I give permission for the footage to be added to an archive of the project that may be added to a public data repository (either University of Plymouth or UK data service) in order that it may be used by other research projects.

## 4 Results

### 4.1 Taxonomy Development (Objective 1)

Following the initial rounds of ecological video analysis, we developed a taxonomy of anxious tells. The taxonomy was constructed not as a static checklist but as a flexible framework rooted in contextual meaning, allowing for the layered and situational nature of affective expression in clinical encounters.

Behaviours were grouped into four overlapping domains:

1. **Embodied Movements** These included involuntary or semi‐voluntary physical movements, often subtle, such as:
  a. Foot tapping, leg bouncing, or ankle rotations beneath the chair
  b. Clenching and unclenching of hands or fingers
  c. Shoulder tension and upper‐body rigidity
  d. Rapid shifts in seated posture or retraction away from the dentist's hands or tools
  e. Tension in the chin and neck area
2. **Facial and Ocular Cues** Often fleeting and easily missed in real time, facial expressions offered key insights when reviewed in slow motion or paused frames:
  a. Compression of lips or pursed mouth
  b. Furrowed brows or asymmetrical micro‐expressions
  c. Gaze aversion, repetitive blinking, fixed stare at a single object (e.g., ceiling light or tile)
  d. Micro‐expressions of startle or wincing following specific sounds or tool contact
  e. Chin Movement
3. **Vocalisations and Paralinguistic Signals** Beyond explicit verbal expressions, paralinguistic cues—tone, breath, pitch—played an important role:
  a. Nervous laughter or over‐compensatory humour
  b. Audible breath holding or sudden exhalation
  c. Voice modulation (e.g., higher pitch, slower pace)
  d. Monosyllabic or minimal verbal engagement
4. **Environmental and Interactional Responses** Anxiety was also legible through how patients responded to the **material and social environment**:
  a. Hyper‐focus on ambient elements (e.g., gripping chair arms, staring at ceiling fan, reacting to alarms or drills)
  b. Hesitancy during equipment explanations or consent procedures
  c. Attempts to delay, interrupt, or deflect the start of a procedure (e.g., asking excessive clarifying questions, telling stories)

The items in 1 and 2 were identified both in the analysis of real‐word data and the simulation lab with exception of chin movement and tension in the chin and neck area that we specifically identified through the video simulation. 3 and 4 were identified in the analysis of real‐world data and confirmed in the simulation lab. Importantly, these tells were rarely consistent across patients or procedures. Instead, anxiety was experienced as patterned but emergent, revealing itself through clusters of signals that had to be read relationally, in terms of timing, interaction, and atmosphere.

To avoid over‐pathologising ordinary behaviour, we developed a guiding principle that: *A tell becomes significant not when it simply appears, but when it disrupts or diverges from the flow of the interaction*. For example, a patient fidgeting continuously may not be anxious, but a sudden stilling of the hands following the sound of a drill may be more revealing. These distinctions were further refined through comparative analysis, where similar procedures across patients were compared to identify situational patterns (e.g., greater anxiety cues preceding injections, reduced movement during post‐treatment explanations). These methods may also be a useful source used to train future AI tools to be able to read patient reactions.

### 4.2 Strength and Limitations of Video Ethnography in Capturing Affective Dynamics (Objective 2)

Video ethnography uncovered affective dynamics that were often missed in real time. Micro‐temporal cues—such as brief hesitations, gaze aversion, postural shifts, and micro‐pauses—became interpretable only when viewed relationally, in connection with clinicians' movements, procedural flow, tools, spatial proximity, and ambient noise. Anxiety thus emerged as a distributed, interactional phenomenon rather than an individual trait.

Co‐viewing revealed interpretive pluralism and professional blind spots. Gestures clinicians typically normalised were read by others as signs of withdrawal or overload, exposing how training and time pressure can shape perception. Viewing the footage also elicited embodied and emotional responses among clinicians and researchers, particularly in encounters involving vulnerable patients, highlighting the clinic's affective intensity.

At the same time, video analysis revealed ethical and interpretive limitations. Meaning was contingent on framing, camera placement, and analytic perspective, and video risked over‐interpretation if treated as transparent evidence. Consistent with video reflexive ethnography (VRE) [18], footage functioned as a provocation for reflection rather than proof. These insights informed the anxiety taxonomy and the design of simulation‐based training, discussed in Section Results, 4.

#### 4.2.1 Key Technical Issues Identified During Simulations

During the simulation phase, several technical challenges emerged that impacted data quality and workflow efficiency. Camera overheating, resolution drops, and synchronization difficulties across multiple devices led to delays and data loss. Bluetooth connectivity proved unreliable for triggering recordings remotely, necessitating manual alternatives. Large data volumes from four‐camera setups strained storage capacity and made post‐session editing and file management time‐consuming. File naming inconsistencies and the lack of clear protocols for handling raw footage further complicated data organisation. Additionally, session timing frequently overran expectations due to role‐play execution, equipment setup, and post‐interview logistics. Ethical documentation processes also required refinement, particularly around separating participation and footage‐use consent. Together, these issues emphasised the need for streamlined simulation protocols, simplified camera setups (e.g., using fewer or 360‐degree cameras), improved data management workflows, and more realistic, focused role‐play scenarios.

### 4.3 Development of a Toolkit (Objective 3)

Based on these findings, we developed a practical guide for video ethnography in dental settings, including:

- A modular camera kit list suitable for clinical or educational use.
- Suggested triangulated camera setups to maximise coverage with minimal disruption.
- Protocols for multi‐angle synchronisation and annotation.
- Guidance on participant consent and visual ethics, especially in relation to facial data.

We also prototyped a training interface, allowing viewers to toggle between simultaneous angles, engage in frame‐by‐frame analysis, and annotate observed tells. This interface supports both researcher interpretation and video reflexive debriefing for trainees ‐ an educational method where learners reflect on recorded consultations to enhance empathy and non‐verbal literacy skills.

The toolkit is available in the Appendix.

### 4.4 Ethical Considerations in Simulation

The simulation lab enabled us to explore ethical grey areas in video‐based research. For example, actors portraying anxious patients occasionally reported experiencing genuine discomfort during certain scenarios. This prompted refinements in consent protocols and underscored the importance of post‐simulation debriefing, not just as a learning tool, but as a care practice.

We also developed tiered consent models to give participants control over how their data could be used (e.g., internal analysis only, anonymised publication, open‐access archival), echoing emerging best practices in video ethnography and affective research.

## 5 Ethical Considerations

Ethics in video ethnographic research extend far beyond consent forms or anonymisation protocols. When studying something as intimate and affectively charged as anxiety—particularly within clinical contexts—ethical engagement must be understood as a continuous, situated, and relational process. This section outlines the ethical principles and practices that shaped both phases of this study: the analysis of real‐world footage and the simulation‐based protocol development.

Ethical procedures were revised in direct response to PPIE input, particularly concerns around visibility, vulnerability, and data control. This led to the implementation of tiered, revisable consent, allowing participants to opt into different levels of recording and use over time, and framing consent as an ongoing process rather than a one‐off transaction. We conceptualise this approach as a form of procedural justice that foregrounds participant agency and data sovereignty.

### 5.1 Consent as Ongoing Relational Practice

Consent in this project was treated not as a single contractual moment but as a layered and revisable process, responsive to the evolving emotional and representational implications of being recorded and analysed. For the simulation lab phase, we developed a three‐tiered consent model that allowed participants (including clinicians and simulated patients) to determine the level of data use they were comfortable with:

- Internal use only (analysis within the research team)
- Anonymised academic dissemination
- Open‐access or public‐facing media use

This model ensured participant agency and control, accommodating shifts in comfort over time. In co‐viewing sessions, participants could review footage, withdraw particular sequences, or revise their permissions—a form of video‐informed consent that mirrors best practices in participatory and visual ethnography.

### 5.2 Interpreting the Body Without Violating It

Video footage, especially close‐up and high‐resolution recordings, has the capacity to reveal what participants may not be aware of—micro‐expressions, flinches, gaze shifts, or tension. While this is methodologically valuable, it raises critical ethical concerns about the right not to be interpreted. We approached every interpretation with epistemic humility, recognising the gap between what is seen and what is felt, and avoiding definitive claims about a participant's internal state.

When interpreting tells of anxiety, we framed behaviours as suggestive rather than diagnostic, and emphasised contextual, relational readings over reductive labelling. This aligns with our ecological‐relational methodology, which views anxiety as emergent, not as an intrinsic trait to be ‘read off’ a body.

### 5.3 Emotional Safety in Simulation

Simulation, while often viewed as a safe pedagogical space, can elicit real affect—especially when simulating vulnerability, pain, or trauma. In our study, simulated patients sometimes described experiencing unexpected emotional discomfort, prompting us to embed structured debriefings after each session. These debriefings functioned both as emotional check‐ins and interpretive dialogues, allowing participants to voice concerns, offer feedback, and reflect on their own reactions.

Moreover, we acknowledged the emotional labour of clinicians—especially those revisiting their own actions during video analysis. Several participants reported discomfort when confronted with missed cues or subtle signs of patient anxiety they had not recognised in the moment. Rather than framing these instances as failures, we approached them as opportunities for compassionate learning and empathic growth. In a different project [19], the researchers implemented a different approach called ‘resolution drop’ protocol—an adaptive strategy that allowed participants to temporarily reduce the emotional fidelity of the simulation when feeling overwhelmed. Much like how a camera lowers resolution under strain, this intentional modulation helped maintain engagement while preventing emotional flooding, ensuring psychological safety during reflective practice.

### Visual Ethics and Data Sovereignty

Given the sensitivity of facial data, our protocol included measures to protect visual privacy, such as:

- Avoiding unnecessary zoom or cropping that accentuated identifiable features.
- Offering pixelation or selective editing upon request.
- Enabling participant withdrawal at any stage of editing or publication.

We also engaged in ongoing discussions around data sovereignty, especially regarding open‐access repositories. While publicly archiving video footage can advance transparency and reuse, it must be balanced against the risks of exposure, misinterpretation, or loss of context. As such, no footage was released without explicit, context‐aware participant permission.

Our ethical stance was rooted in the belief that affective research requires affective care towards participants, towards data, and towards the interpretive process itself. Rather than seeing ethics as a boundary to creativity or rigour, we approached it as a generative force that shaped every methodological decision and analytical insight.

## 6 Discussion

This study set out to explore how dental anxiety manifests as a dynamic, relational, and embodied phenomenon, and to examine how video ethnography, enhanced by ecological and affective theory, can help make such phenomena visible, interpretable, and actionable in both research and clinical training. Across both the real‐world and simulation phases, our findings highlight the rich potential of video as a sensory, spatial, and affective tool for understanding patient experience and enhancing practitioner sensitivity. Importantly, PPIE in this study functioned not merely as consultation but as a form of co‐production that shaped methodological boundaries. Contributors helped define what forms of visibility were acceptable, when recording shifted from supportive to intrusive, and how vulnerability should be handled across different patient groups. This directly influenced how we interpreted anxious tells as relational and situational phenomena, rather than as fixed indicators, and reinforced the need to read behavioural and physiological cues in context rather than as linear signals of anxiety.

### 6.1 Rethinking Dental Anxiety: From Trait to Terrain

One of the central contributions of this study is the reframing of dental anxiety not as a stable, internal ‘trait’ that resides within the patient, but as a terrain of affect that emerges through micro‐interactions, clinical rhythms, spatial arrangements, and interpersonal atmospheres. This resonates with affect theory, particularly Raymond Williams' concept of *structures of feeling*, which emphasises the importance of lived, collective, and often pre‐conscious experience.

Rather than reducing anxiety to discrete symptoms or verbal reports, our ecological‐relational approach reveals how it is distributed across bodies, spaces, and tools, and how it evolves over time. This re‐conceptualisation diverges from many prevailing models in both psychology and dental education that treat affect as measurable, linear, and individualised. This transition from perceiving anxiety as a fixed characteristic to acknowledging it as a relational context illustrates what Meyer and Land [11] describe as ‘troublesome knowledge’, a conceptual shift, that is, initially counterintuitive and necessitates practitioners to abandon established frameworks. The video‐based taxonomy created here is not a diagnostic checklist; instead, it is a tool for helping practitioners find their way through this threshold. It helps them develop new ways of seeing that are based on embodied, situated cognition [20] instead of just recognising symptoms out of context.

Our findings align closely with Höglund et al.'s [21] grounded theory model in which the ‘clinical eye’ functions as the core process through which clinicians recognise dental anxiety, supported by categories including sympathetic activation, patient‐reported anxiety, controlling behaviours, avoidance, and accomplishment. While Höglund et al. describe what clinicians attend to, our ecological video analysis makes these processes visible at the micro‐interactional level, translating the framework into a taxonomy of observable anxious tells embedded in posture, breath, gaze, timing, and interactional flow. Importantly, our categorisation does not replace clinical judgement but supports it, offering a structured way to reflect on the same relational cues that Höglund identifies as underpinning expert recognition.

### 6.2 Beyond Communication: Attunement and Atmosphere

While communication skills training in dentistry often focuses on improving verbal exchanges and building rapport, our findings suggest that non‐verbal literacy and atmospheric awareness are equally vital. Clinicians often responded intuitively to signs of discomfort, slowing down, softening tone, pausing procedures, but such responses were not always conscious or consistent. This points to the need for pedagogical tools that train attentiveness, not only skill. This has implications for improving patient care while also reducing clinician burnout, which is often exacerbated by emotionally charged encounters where discomfort goes unrecognised or unaddressed.

### 6.3 Video as Provocation and Pedagogy

We learned that to capture what we sense as unplanned incidentals, as in this project, the devices have to be used in ways that are not always consistent with their mainstream or even intended design. This applies not only to positioning but also to other technical considerations such as lighting and depth of field for example. This attention to creativity also invites a constant experimental and inventive use of the technology in collaboration with the research processes as they develop. It also suggests that in this ‘experimental’ mode there is a powerful role for video reflexive ethnography (VRE) in dental education, not just as a tool for assessment, but as a site of care, reflection, and transformation. Importantly, video's ability to slow time, amplify detail, and present multiple perspectives can cultivate a valuable form of visual literacy that is often neglected in clinical training.

### 6.4 Designing for Affect: From Simulation to Implementation

The simulation lab phase of this study enabled us to refine the technical and ethical dimensions of video‐based methods. We developed practical guidelines for camera setup, multi‐angle recording, and consent templates, which can be adopted or adapted in real‐world clinics, simulation centres, or training programmes. These protocols are not only technical artefacts; they are designed to support positive affective response, to make the unseen visible, the subtle interpretable, and the relational recordable.

In parallel, we prototyped a framework for machine‐vision‐informed anxiety detection, guided by the human‐coded taxonomy of tells. While preliminary, this work gestures toward future systems in which AI tools are not used to replace human judgment but to enhance clinical awareness and trigger reflective dialogue. However, this demands critical attention to issues of interpretability, bias, and contextual fidelity.

### 6.5 Limitations and Future Research

This study had several limitations. The original footage was not filmed with anxiety as its primary focus, meaning that some camera angles or clinical scenarios were suboptimal for affective interpretation. Moreover, while our taxonomy was grounded in rich qualitative analysis, its generalisability across cultural contexts, age groups, and clinical settings remains to be tested.

Future work should include:

- Triangulation with biometric and patient‐reported data, to correlate behavioural tells with physiological and/or subjective markers.
- Longitudinal studies to explore how clinicians' awareness of anxiety evolves through exposure to video‐reflexive practices.
- Expansion into other emotionally charged clinical settings, such as paediatrics, palliative care, or emergency medicine.

Ultimately, this project confirms that patient anxiety in dentistry is not just something to be managed, it is something to be understood, witnessed, and worked with, both relationally and aesthetically. Using video ethnography in this way that we did allows us to reimagine the clinic as a site of affective choreography and the camera as a tool of care. It shows a pathway to a new language and a new practice for cultivating positive affect responses and insight in clinical life.

Future research could extend this ecological–relational approach by integrating physiological and other multimodal measures alongside video ethnography, not as a ‘ground truth’ for anxiety but as another situated modality in the affective ecology of care. In dental settings, heart rate and blood pressure have been used as clinical parameters associated with dental anxiety and its temporal dynamics across procedures, and heart‐rate variability (HRV) has been explored as a real‐time indicator of autonomic activity during anxiety‐provoking treatment contexts such as third‐molar surgery [22]. Similarly, cardiovascular responses have been reported around local anaesthetic delivery, where anticipation, pain, age, and traumatic history can interact with arousal patterns [23]. These strands suggest that multimodal datasets (e.g., HR/HRV, skin conductance, respiration) could enrich future work when read in relation to the micro‐choreography of interaction captured on video.

Crucially, however, our framework resists linear biomedical inference (e.g., ‘increased HR = dental anxiety’). Both psychophysiological traditions and clinical realities caution that autonomic changes can be multiply determined and context‐dependent, shaped by broader life circumstances and concurrent stressors. This was also evident in our early stakeholder engagement: participants described situations where a patient arrived in clinic already physiologically activated (‘a bad day’), meaning elevated heart rate could reflect distress unrelated to dentistry (e.g., prior events, pain, fatigue, interpersonal stress), and only becomes interpretable when timed against the dental stressor and read alongside behaviour, talk, and environment. In this sense, physiology should be analysed as part of the field, not a decontextualised signal; multimodal triangulation here is about thickening interpretation, not validating a hidden internal state.

Methodologically, advances in contact‐free sensing (e.g., video‐based heart‐rate estimation via facial signal extraction [24]) may allow physiological layers to be incorporated without adding burden or disrupting clinical flow, aligning well with ecological video research and simulation‐based camera testing. Taken together, a next step is to develop a multimodal protocol that synchronises video‐observed tells, patient‐reported experience, and physiological rhythms, while explicitly modelling ambiguity, competing explanations, and the wider context in which arousal emerges.

## 7 Conclusion

This study has introduced an ecological‐relational approach to video ethnography for exploring dental anxiety, reframing it as a dynamic, situational phenomenon rather than a fixed trait. By analysing real‐world dental procedures and testing camera protocols in simulation, we developed a context‐sensitive taxonomy of anxious tells and demonstrated how video can be used not just to observe but to provoke reflection, positive affect, and transformation in dental training.

We found that anxiety is best understood not in isolation, but as co‐produced by interactions, atmospheres, rhythms, and environmental factors. The video camera—when carefully positioned and ethically used—can reveal this complexity, offering a medium through which the emotional life of clinical practice becomes more visible and discussable.

We propose that video reflexive methods, grounded in sensory and affective theory, can play a central role in reshaping how dentists are trained to notice, interpret, and respond to patient distress. Beyond technical skills, this work invites the cultivation of attentive presence ‐ a way of seeing, listening, and being with patients that acknowledges anxiety as relational and responsive. The latter can be used in training future dentists.

In doing so, we contribute a methodological and ethical foundation for future research, simulation design, and potentially AI‐assisted tools that can support emotionally intelligent, anxiety‐aware care.

## Author Contributions

**Mona Nasser:** conceptualization, investigation, funding acquisition, writing – original draft, methodology, validation, writing – review and editing, formal analysis, project administration, data curation, supervision, resources. **Hannah Drayson:** conceptualization; methodology; writing – review and editing. **Sally Hanks:** conceptualization, writing – review and editing. **Nicholas Peres:** conceptualization, methodology, writing – review and editing. **Michael Punt:** Conceptualization, funding acquisition, writing – review and editing, methodology, supervision.

## Conflicts of Interest

The authors declare no conflicts of interest.

## Data Availability

The data that support the findings of this study are available on request from the corresponding author. The data are not publicly available due to privacy or ethical restrictions. Due to the sensitive nature of data, the data cannot be made available publicly.

## Step‐by‐Step Guidance for Dentists to Use Cameras for Recording Dental Sessions

### 1

This guide provides practical steps for dentists to use cameras effectively for various purposes, including legal documentation, training, and facilitating discussions with patients to improve their dental experience.

1. **Practice Evaluation**
  - Identify all light sources in the dental practice, including overhead lights, windows, and reflective surfaces.
  - Evaluate potential blockages in the practice, such as dental equipment and staff movements, that may obstruct camera angles.
  - Observe and document the typical movement patterns of staff during procedures to predict areas prone to visual obstructions.
  - Ensure that the placement of the dental chair allows for optimal camera coverage without sacrificing the comfort of the patient or the efficiency of the dental team.
2. **Equipment Evaluation**
  - Choose a camera suited for the intended purpose (e.g., a wide‐angle lens for general coverage, a close‐up lens for detailed work).
  - Check the camera's field of view and ensure it is wide enough to capture essential details.
  - Evaluate battery life to ensure the camera can record for the duration of the session without interruption.
  - Select a camera with connectors compatible with your practice's setup for easy data transfer.
  - Consider using cameras with protective casings to shield them from aerosols or other contaminants generated during procedures.
3. **Informed Consent**
  - Develop a clear and transparent consent process. Provide patients with multiple options for how their data may be used (e.g., legal documentation, training, research, or patient‐specific debriefing).
  - Share a Patient Information Sheet (PIS) with detailed explanations of how recordings will be stored, shared, or anonymized, depending on the purpose.
  - Allow patients to make informed decisions and document their consent appropriately before proceeding with recordings.
  - Highlight that patients can withdraw their consent at any time before or after the recording.
4. **Testing Shots with Actors in a Simulated Environment**
  - Conduct mock procedures with actors to replicate typical movements and interactions in the dental setting.
  - Test camera placements under realistic conditions, accounting for staff movements, patient positioning, and equipment usage.
  - Use these simulations to refine the camera setup and identify areas for improvement in capturing critical data.
5. **Identifying Ideal Angles to Capture Non‐Verbal Cues**
  - Optimize camera angles to capture the taxonomy of anxiety indicators, including hand movements, chin tension, posture, and other non‐verbal cues.
  - Use a combination of close‐up, mid‐range, and wide‐angle shots to ensure all relevant behaviours are recorded.
  - Avoid placing cameras in positions prone to obstruction by equipment or staff.
6. **Presenting Recordings to Patients**
  - For training purposes: Use synchronised multi‐angle footage to provide a comprehensive view of patient‐dentist interactions. This approach allows for forensic‐style analysis of anxiety indicators and behaviours, but it requires editing expertise to synchronise multiple camera views effectively.
  - For post‐treatment discussions with patients: Use a single wide‐angle shot to facilitate conversations. This approach simplifies the presentation and focuses on improving the patient's comfort and experience during future dental visits.
  - Ensure that the presentation format of the visual material aligns with the purpose (e.g., detailed multi‐shot analysis for training vs. simplified single wide‐shot for patient feedback).

By following these steps, dentists can use cameras effectively to meet a variety of needs, from improving patient experiences to facilitating staff training and ensuring legal compliance.
